# Research Progress of Sarcopenia Index in Chronic Obstructive Pulmonary Disease: A Review

**DOI:** 10.7759/cureus.97556

**Published:** 2025-11-23

**Authors:** Xiaolong Li, Shuhao Xu, Chunfang Zeng, Xin Wang, Rongli Wang, Yinhe Feng

**Affiliations:** 1 Pulmonary and Critical Care Medicine, Deyang People's Hospital, Deyang, CHN; 2 Stomatology, Deyang People's Hospital, Deyang, CHN; 3 Pulmonary and Critical Care Medicine, The Affiliated Hospital of Southwest Medical University, Luzhou, CHN

**Keywords:** chronic obstructive pulmonary disease, clinical outcomes, disease severity, exercise capacity, lung function, sarcopenia index, skeletal muscle

## Abstract

Sarcopenia, characterized by the decline in skeletal muscle mass and function, has emerged as a prevalent syndrome among patients with chronic obstructive pulmonary disease (COPD), significantly affecting their quality of life and clinical outcomes. The sarcopenia index, an essential tool for assessing sarcopenia, has recently gained attention for its potential role in the diagnosis, disease evaluation, and therapeutic monitoring of COPD. This review summarizes the definition and measurement methods of the sarcopenia index and highlights its application progress in COPD management. We focus on the impact of sarcopenia on lung function, exercise capacity, quality of life, disease severity, and clinical prognosis in COPD patients. Additionally, we discuss current research on intervention strategies targeting sarcopenia and explore future directions to improve COPD patient care through the integration of sarcopenia assessment. By providing a comprehensive overview, this article aims to deepen understanding of the sarcopenia index’s significance and encourage further research in this evolving field.

## Introduction and background

Chronic obstructive pulmonary disease (COPD) is a prevalent and progressive respiratory disorder characterized by persistent airflow limitation and chronic respiratory symptoms such as cough, sputum production, and dyspnea. It represents a significant global health burden, ranking among the leading causes of morbidity and mortality worldwide [1,2]. The diagnosis of COPD is confirmed by spirometry demonstrating a post-bronchodilator forced expiratory volume in one second (FEV1)/forced vital capacity (FVC) ratio less than 0.7, with severity classified according to the Global Initiative for Chronic Obstructive Lung Disease (GOLD) criteria [1]. The disease course is characterized by periods of stability and acute exacerbations, which contribute to accelerated lung function decline and increased mortality risk [3,4]. COPD is associated with systemic inflammation, oxidative stress, and a spectrum of comorbidities, including cardiovascular diseases, metabolic syndrome, osteoporosis, depression, and notably, skeletal muscle dysfunction [1,5,6]. Comorbid conditions are highly prevalent in COPD. Epidemiological data show that approximately 26% of patients have concurrent type 2 diabetes [7]. Furthermore, Chen et al. documented a 59.6% prevalence of comorbid coronary artery disease (CAD) [8]. Evidence from a large-scale review of 8,753 individuals indicates a global pooled prevalence of osteoporosis of 38% [9]. Notably, depressive symptoms were identified in 49.7% of a 2,921-patient cohort [10]. The reported prevalence of skeletal muscle dysfunction exhibits considerable heterogeneity, ranging from 15% to 50%, likely attributable to methodological differences among studies [11]. These comorbidities substantially worsen patients’ health status, impair quality of life, and complicate disease management [12,13].

Among the systemic manifestations of COPD, skeletal muscle dysfunction and sarcopenia have emerged as critical contributors to disease burden. Sarcopenia, defined as the progressive loss of skeletal muscle mass and strength, is highly prevalent in COPD patients, with reported prevalence rates ranging from approximately 15% to over 50% depending on diagnostic criteria, disease severity, and study populations [14-16]. The pathophysiology of sarcopenia in COPD is multifactorial, involving chronic systemic inflammation, oxidative stress, hypoxia-induced metabolic alterations, physical inactivity, nutritional deficiencies, hormonal imbalances, and mitochondrial dysfunction [13,17,18]. These factors interact to promote muscle protein catabolism, impair muscle regeneration, and induce a shift from oxidative (type I) to glycolytic (type II) muscle fibers, resulting in reduced muscle endurance and strength [15,17]. Importantly, sarcopenia in COPD is associated with poor clinical outcomes, including reduced exercise capacity, increased risk of acute exacerbations, higher hospitalization rates, impaired quality of life, and increased mortality [19-21].

The clinical relevance of sarcopenia in COPD has been increasingly recognized, prompting efforts to develop reliable assessment methods and biomarkers to facilitate early identification and monitoring. Traditional diagnosis of sarcopenia requires assessment of muscle mass, muscle strength, and physical performance, typically involving dual-energy X-ray absorptiometry (DXA), bioelectrical impedance analysis (BIA), handgrip strength measurement, and gait speed tests [21,22]. However, these methods may be limited by accessibility, cost, or patient factors such as respiratory impairment. Recent studies have explored the utility of computed tomography (CT)-based measurements of muscle cross-sectional area, particularly of the rectus femoris or erector spinae muscles, as objective and reproducible indices of muscle mass in COPD [23-25]. Ultrasound assessment of muscle thickness and cross-sectional area has also shown promise as a noninvasive bedside tool for sarcopenia screening [26]. Furthermore, circulating biomarkers such as growth differentiation factor-15 (GDF15), C-terminal agrin fragment (CAF), neurofilament light chain (NfL), and inflammatory indices have been investigated for their correlation with muscle mass, strength, and sarcopenia status in COPD patients [16, 27-29].

Sarcopenia is not only a comorbidity but also a treatable trait in COPD, amenable to interventions targeting muscle mass and function. Pulmonary rehabilitation programs incorporating exercise training remain the cornerstone of sarcopenia management, improving muscle strength, exercise tolerance, and quality of life [18,30]. Nutritional support, including protein supplementation, vitamin D, omega-3 fatty acids, and antioxidants, complements exercise by addressing malnutrition and systemic inflammation often present in COPD [13,31,32]. Emerging evidence supports the use of pharmacological agents and novel therapies targeting molecular pathways involved in muscle wasting, although these remain largely experimental [18,33]. Multidisciplinary approaches integrating pulmonology, nutrition, physiotherapy, and geriatrics are essential for optimal management [18,34].

Despite advances, gaps remain in understanding the precise molecular mechanisms linking COPD and sarcopenia, the standardization of diagnostic criteria, and the development of personalized interventions. Recent scientometric analyses highlight the growing research interest in COPD-associated sarcopenia, emphasizing the need for further studies on pathophysiology, diagnostic tools, and therapeutic strategies [35]. Given the substantial impact of sarcopenia on COPD prognosis and healthcare burden, systematic evaluation and targeted management of muscle dysfunction should be integrated into routine COPD care.

In summary, COPD is a chronic respiratory disease with high global prevalence and mortality, complicated by frequent comorbidities including sarcopenia. Sarcopenia significantly worsens clinical outcomes by impairing muscle mass and function, thereby reducing exercise capacity and quality of life. The sarcopenia index, incorporating muscle mass and functional assessments, has gained attention as a composite marker to evaluate skeletal muscle status in COPD patients. This review aims to summarize the current research progress on sarcopenia indices in COPD, exploring their clinical application value and guiding future research directions to improve patient management and outcomes.

## Review

The definition and measurement methods of sarcopenia index

Definition and Classification Criteria of Sarcopenia Index

Sarcopenia is a complex clinical syndrome characterized by a progressive and generalized loss of skeletal muscle mass, muscle strength, and physical function, leading to adverse outcomes such as frailty, falls, disability, and mortality [36,37]. The definition of sarcopenia has evolved from focusing solely on muscle mass to encompassing muscle strength and physical performance, reflecting the understanding that muscle quality and function are as critical as muscle quantity [36,38]. International consensus groups, such as the European Working Group on Sarcopenia in Older People (EWGSOP) and the Asian Working Group for Sarcopenia (AWGS), have developed diagnostic criteria integrating these components. The EWGSOP2 criteria emphasize low muscle strength as the primary parameter, confirmed by low muscle quantity or quality, and severe sarcopenia is diagnosed when physical performance is also impaired [39]. Similarly, AWGS 2019 criteria provide region-specific cutoffs for muscle mass, strength, and function, tailored to Asian populations [40,41]. In COPD patients, sarcopenia has particular significance due to its association with worse clinical outcomes, including increased mortality and hospitalization [42,43]. However, the heterogeneity of COPD and the influence of comorbidities necessitate careful application of sarcopenia indices in this population [44]. The sarcopenia index in COPD often requires integration of muscle mass, strength (e.g., handgrip strength), and physical performance (e.g., gait speed or 6-minute walk test) to accurately reflect the patient’s functional status and prognosis [43,45]. Moreover, sarcopenia in COPD may be complicated by systemic inflammation and nutritional deficiencies, further underscoring the need for comprehensive assessment [43,46]. Thus, the sarcopenia index is a multidimensional construct combining muscle mass, strength, and functional capability, with classification standards provided by EWGSOP and AWGS, adapted for specific populations such as COPD patients to guide clinical management and risk stratification.

Common Measurement Techniques of Sarcopenia Index

The assessment of sarcopenia index involves quantifying skeletal muscle mass, muscle strength, and physical function using various measurement techniques. For muscle mass, imaging modalities such as dual-energy X-ray absorptiometry (DXA), bioelectrical impedance analysis (BIA), magnetic resonance imaging (MRI), and computed tomography (CT) are commonly employed [38,47]. DXA is widely used due to its accessibility, low radiation dose, and established cutoffs for appendicular skeletal muscle mass, although it provides limited information on muscle quality [45,48]. BIA offers a portable and cost-effective alternative but may be influenced by hydration status and other factors [49]. MRI and CT provide detailed assessments of muscle mass and composition, including fat infiltration, but are less accessible and involve higher costs or radiation exposure [46,50]. In COPD patients, CT-based skeletal muscle index (SMI) at the lumbar vertebrae level is frequently used to evaluate muscle mass and predict outcomes [45,51]. Muscle strength is primarily measured by handgrip strength using dynamometers such as the Jamar, Grip-D, or newer devices validated for reliability and accuracy [52]. Handgrip strength correlates with overall muscle strength and functional capacity in COPD and sarcopenia [53,54]. Physical function is assessed through performance tests including gait speed over short distances (four or six meters), the 6-minute walk test (6MWT), timed up and go (TUG), and chair stand tests [55,56]. These tests reflect endurance, mobility, and balance, which are often impaired in sarcopenia and COPD [57,58]. A consistent and independent inverse association has been established between the 6MWD and sarcopenia in COPD. As such, the severity of sarcopenia (reflected by diminished muscle mass, strength, and physical performance) is a key determinant of a reduced 6MWD and impaired exercise capacity [59-61]. Consequently, the 6-minute walk test (6MWT) serves not only as a measure of functional exercise capacity but also as a valuable and practical clinical marker for the presence of systemic muscle dysfunction characteristic of sarcopenia. Recent advances include the use of wearable sensors and machine learning to quantify gait parameters and muscle function remotely [62,63]. Combining these modalities allows a comprehensive evaluation of sarcopenia index, capturing muscle quantity, strength, and functional performance critical for diagnosis and management in COPD patients.

Clinical Application and Evaluation Standards of Sarcopenia Index

Clinically, the sarcopenia index integrates muscle mass, strength, and physical function to diagnose sarcopenia and stratify patients for management, particularly in chronic diseases like COPD [42,64]. The EWGSOP2 and AWGS guidelines provide evaluation standards, recommending initial screening (e.g., SARC-F questionnaire), followed by assessment of muscle strength (handgrip strength), muscle mass (DXA, BIA, or CT), and physical performance (gait speed, 6MWT, or TUG) [39,41]. Thresholds for these parameters are population-specific; for example, AWGS 2019 provides cutoffs adapted for Asian populations, while European criteria differ [40,65]. In COPD patients, sarcopenia index thresholds may require adjustment due to disease-related muscle alterations and comorbidities [50,64]. The sarcopenia index aids in prognostication, as sarcopenia is associated with increased mortality, hospitalization, and poor functional outcomes in COPD [42,43]. Evidence indicates that sarcopenia is negatively associated with the risk of respiratory failure and severe pneumonia in COPD patients [66]. Furthermore, a study by Dai [24] et al., which enrolled 148 patients hospitalized in the intensive care unit for acute exacerbation of COPD, compared outcomes between sarcopenic and non-sarcopenic groups. The study found that the sarcopenic group had significantly higher rates of both 1-year and overall COPD-related mortality, as well as all-cause mortality. Multivariate Cox regression analyses revealed sarcopenia as a risk factor for 1-year (HR = 3.981 [1.137-13.938], p = 0.031) and overall (HR = 2.308 [1.310-4.065], p = 0.004) mortality. Moreover, the sarcopenia index informs therapeutic decisions, including nutritional support, resistance exercise, and pharmacological interventions [67,68]. Different studies have proposed varying cutoffs for sarcopenia index components, reflecting heterogeneity in measurement techniques and populations [69,70]. Thus, clinical application requires careful selection of assessment methods and thresholds, considering patient demographics and disease context. The sarcopenia index supports risk stratification and individualized management in COPD, emphasizing the importance of comprehensive evaluation combining muscle mass, strength, and physical function according to established international standards.

Correlation analysis between sarcopenia Index and COPD

Epidemiological Characteristics of Sarcopenia in COPD Patients

Sarcopenia is a prevalent extrapulmonary manifestation in patients with COPD, with its epidemiological characteristics varying across populations and disease severities. Meta-analyses and systematic reviews report a wide prevalence range of sarcopenia in COPD patients, from approximately 8% in population-based cohorts to over 60% in nursing home residents, with an average prevalence of around 20-30% [71-73]. This variability is partly attributable to differences in diagnostic criteria, measurement methods (e.g., bioelectrical impedance analysis, dual-energy X-ray absorptiometry), and patient demographics. Notably, sarcopenia prevalence increases with COPD severity; patients classified as GOLD stage 3 or 4 exhibit significantly higher rates of sarcopenia compared to those with milder disease [74,75]. Gender differences have also been observed, with male COPD patients showing a higher prevalence of sarcopenia than females [71]. Age is a consistent risk factor, as older COPD patients are more susceptible to muscle mass loss and functional decline [73,76]. Other identified risk factors influencing sarcopenia development in COPD include poor nutritional status, systemic inflammation, oxidative stress, smoking history, and comorbidities such as diabetes mellitus and chronic renal failure [75-78]. Inflammatory markers like interleukin-6 have been linked to sarcopenia presence, suggesting that systemic inflammation plays a pivotal role [79]. Additionally, oxidative stress biomarkers correlate with muscle wasting and weakness in COPD [78,80]. Reduced physical activity and ventilatory limitations inherent to COPD further exacerbate muscle atrophy [81]. Collectively, these epidemiological data underscore that sarcopenia is highly prevalent in COPD, particularly among elderly patients with advanced disease and multiple risk factors, highlighting the need for routine screening and targeted interventions in this population.

Relationship Between Sarcopenia Index and Pulmonary Function Parameters in COPD

The sarcopenia index, reflecting muscle mass and function, is intricately linked with pulmonary function parameters in COPD patients. Studies consistently demonstrate that sarcopenia correlates with reduced forced expiratory volume in one second (FEV1) and FEV1/FVC ratios, indicating more severe airflow obstruction [75,82]. COPD patients with sarcopenia exhibit poorer spirometric indices compared to non-sarcopenic counterparts, and the degree of muscle wasting is inversely associated with lung function [74,83]. Mechanistically, systemic inflammation, oxidative stress, and nutritional deficiencies contribute to both muscle degradation and pulmonary impairment [78,79]. Sarcopenia exacerbates respiratory muscle weakness, leading to diminished maximal inspiratory and expiratory pressures, which further compromise ventilatory capacity [80,84]. Moreover, nocturnal hypoxemia, common in COPD, has been linked to sarcopenia via muscle atrophy pathways, suggesting hypoxia-induced muscle loss as a contributing factor to pulmonary function decline [85]. The sarcopenia index also predicts the rate of lung function deterioration, with sarcopenic patients experiencing accelerated decline in FEV1 over time [75]. A prospective study by Wu [5] et al. enrolled 120 elderly COPD patients, of whom 63 (52.5%) were diagnosed with sarcopenia. During the one-year follow-up period, the sarcopenic group demonstrated significantly poorer lung function compared to the non-sarcopenic group. Multivariate analysis confirmed that the presence of sarcopenia was significantly associated with a lower FEV1 in these patients (OR = 0.97, 95% CI: 0.94-1.00, p = 0.035). Imaging biomarkers such as the sarcopenia index derived from chest CT scans correlate with pulmonary function and can prognosticate disease progression [24]. Overall, these findings indicate that sarcopenia is not merely a comorbidity but actively influences the severity of airway obstruction and the trajectory of lung function decline in COPD patients.

Impact of Sarcopenia Index on Exercise Capacity and Quality of Life in COPD Patients

Sarcopenia significantly impairs exercise capacity and quality of life in COPD patients, with the sarcopenia index serving as a critical determinant of functional status. Reduced muscle mass and strength, as measured by the sarcopenia index, correlate with decreased six-minute walk distance (6MWD), indicating diminished exercise tolerance [75,84]. COPD patients with sarcopenia demonstrate lower gait speed and handgrip strength, both of which are associated with poorer physical performance and endurance [27,74]. The decline in muscle function contributes to increased dyspnea and fatigue, exacerbating limitations in daily activities [74]. Quality of life assessments using COPD-specific tools such as the COPD Assessment Test (CAT) and the modified Medical Research Council (mMRC) dyspnea scale reveal worse scores in sarcopenic patients, reflecting greater symptom burden and functional impairment [19,75]. Furthermore, sarcopenia is linked to a higher frequency of acute exacerbations and hospitalizations, which negatively affect patients’ health-related quality of life and increase healthcare utilization [19,86]. The presence of sarcopenia also correlates with systemic inflammation and altered myokine profiles, which may mediate muscle dysfunction and reduced exercise capacity [84]. Interventions targeting muscle mass preservation, including exercise training and nutritional supplementation, have shown potential in improving exercise tolerance and quality of life in sarcopenic COPD patients [81]. Thus, the sarcopenia index is a valuable marker for identifying COPD patients at risk for functional decline and diminished quality of life.

Influence of Sarcopenia Index on Disease Severity and Clinical Outcomes in COPD Patients

Sarcopenia and the sarcopenia index are closely associated with increased disease severity and adverse clinical outcomes in COPD patients. Sarcopenic COPD patients exhibit higher rates of respiratory failure and are more likely to require non-invasive or invasive mechanical ventilation during acute exacerbations [86]. The presence of sarcopenia independently predicts all-cause mortality and COPD-related mortality, with sarcopenic patients demonstrating significantly higher death rates compared to non-sarcopenic individuals [19,86,87]. Sarcopenia also contributes to a hypercoagulable state, which may exacerbate disease progression and complications [86]. Longitudinal studies reveal that sarcopenia is associated with increased frequency and severity of acute exacerbations, leading to more frequent hospital admissions and intensive care unit stays [5,19]. Imaging-based sarcopenia indices, such as skeletal muscle index measured by chest CT, have prognostic value in predicting long-term mortality in critically ill COPD patients [24]. Furthermore, sarcopenia is linked with systemic immune-inflammation indices, which independently increase mortality risk in COPD [87]. These findings underscore the importance of early detection and management of sarcopenia to improve clinical outcomes and reduce mortality in COPD patients.

The application and future prospects of sarcopenia index in COPD management

Sarcopenia Index-Guided Individualized Treatment Strategies for COPD Patients

The sarcopenia index, reflecting the degree of skeletal muscle loss and dysfunction, plays a critical role in guiding individualized treatment strategies for patients with COPD. Nutritional support and rehabilitation exercise programs are foundational components of managing sarcopenia in COPD, as muscle wasting significantly impacts patients’ functional status and prognosis. Evidence indicates that sarcopenia is prevalent in COPD and correlates with disease severity, reduced exercise capacity, and increased mortality risk [19,74]. Nutritional interventions tailored to sarcopenia index levels focus on correcting malnutrition and providing adequate protein and energy intake to counteract muscle catabolism. For instance, nutritional supplementation with essential amino acids, omega-3 fatty acids, vitamin D, and antioxidants has been shown to improve respiratory function and muscle strength in COPD patients [32,88]. Rehabilitation exercises, particularly resistance and endurance training, are designed based on the sarcopenia index assessment to enhance muscle mass and function, thereby improving dyspnea scores and exercise tolerance [19,89]. Moreover, the application of ultrasound assessment of the rectus femoris muscle offers a noninvasive method to monitor sarcopenia and adjust rehabilitation protocols accordingly [26].

Pharmacological interventions also play a role in managing sarcopenia within COPD treatment paradigms. Anti-inflammatory drugs and corticosteroids are commonly used in COPD management; however, their effects on muscle mass are complex. Chronic corticosteroid use may exacerbate muscle wasting, necessitating careful balancing of benefits and risks [90]. Novel therapeutic targets are under investigation, including agents that modulate neuromuscular junction integrity and inflammatory pathways. Biomarkers such as C-terminal agrin fragment-22 (CAF22), brain-derived neurotrophic factor (BDNF), and glial cell line-derived neurotrophic factor (GDNF) have been identified as predictors of sarcopenia and may guide pharmacologic strategies during pulmonary rehabilitation [91]. Additionally, elevated plasma glucagon-like peptide-1 (GLP-1) levels have been correlated with sarcopenia severity in elderly COPD patients, suggesting potential avenues for targeted drug development [92]. The integration of the sarcopenia index in clinical practice enables a comprehensive approach that combines nutritional, exercise, and pharmacologic therapies tailored to individual patient profiles, aiming to mitigate muscle loss, reduce acute exacerbations, and improve overall quality of life in COPD patients.

Role of Sarcopenia Index in Prognostic Evaluation of COPD

The sarcopenia index serves as a valuable prognostic biomarker in COPD, providing insight into patients’ mortality risk and likelihood of complications. Multiple studies have demonstrated that sarcopenia, as quantified by muscle mass indices and functional measures, independently predicts adverse clinical outcomes in COPD populations. For instance, sarcopenic COPD patients exhibit higher rates of acute exacerbations, pneumonia, and all-cause mortality compared to non-sarcopenic counterparts [19,87]. The sarcopenia index correlates with established prognostic tools such as the BODE index (body mass index, airflow obstruction, dyspnea, and exercise capacity), further validating its clinical utility [19,74]. Moreover, sarcopenia assessed via computed tomography (CT) muscle measurements at thoracic vertebra levels has been linked to long-term mortality in critically ill COPD patients, underscoring the index’s role in risk stratification [24].

Combining the sarcopenia index with other biomarkers enhances prognostic accuracy. Inflammatory markers such as the neutrophil-to-lymphocyte ratio (NLR), prognostic nutritional index (PNI), and systemic immune-inflammation index have been integrated with sarcopenia assessments to construct comprehensive prognostic models [5,79,93]. For example, COPD patients with both sarcopenia and elevated systemic inflammation indices exhibit significantly increased mortality risk compared to those with either condition alone [87]. Additionally, plasma levels of myokines like BDNF and soluble TNF receptors correlate with functional and respiratory performance, offering further prognostic insights when combined with sarcopenia index [84]. Advanced imaging techniques and artificial intelligence-assisted analysis of chest CT scans are emerging as promising tools for automated sarcopenia quantification and integration with other clinical parameters to refine prognostic models [94].

Overall, the sarcopenia index is a robust predictor of COPD prognosis, particularly when used in conjunction with systemic inflammatory biomarkers and nutritional assessments. Its application facilitates early identification of high-risk patients, enabling timely interventions aimed at reducing exacerbations, hospitalizations, and mortality. Future research focusing on standardized sarcopenia index measurement protocols and validation of integrated prognostic models will further enhance its clinical applicability in COPD management.

## Conclusions

In conclusion, the integration of sarcopenia indices as a pivotal tool for assessing skeletal muscle status in patients with COPD represents a significant advancement in both clinical evaluation and management of this complex disease. From an expert perspective, the development of sarcopenia assessment methodologies has enhanced our understanding of the multifaceted impact of muscle wasting on COPD progression, functional decline, and overall patient prognosis. The evidence consistently highlights a high prevalence of sarcopenia among COPD patients, establishing it not only as a common comorbidity but also as a critical determinant of disease severity, exercise capacity, quality of life, and mortality risk. Balancing the diverse research perspectives, it is clear that while pulmonary function tests remain the cornerstone of COPD evaluation, the incorporation of muscle mass and function assessments through sarcopenia indices provides a more comprehensive picture of patient health. This multidimensional approach acknowledges the systemic nature of COPD, moving beyond the lungs to address muscle dysfunction that significantly contributes to patient morbidity. The challenge lies in harmonizing these findings to develop standardized, reproducible measurement protocols that can be widely adopted in clinical practice. Such standardization is essential to ensure consistency in diagnosis, facilitate comparative research, and guide targeted interventions.

Moreover, the application of sarcopenia indices enables individualized treatment planning and prognostic evaluation, which are crucial for optimizing COPD management strategies. Tailoring interventions to address muscle loss-whether through nutritional support, physical rehabilitation, or pharmacological therapies-can potentially mitigate the decline in exercise tolerance and improve patient outcomes. This personalized approach aligns with the broader trend in medicine toward precision health, where treatment is adapted to the unique characteristics of each patient. Looking forward, there is a pressing need to intensify research efforts focused on refining sarcopenia assessment tools and validating their clinical utility across diverse COPD populations. Currently, the diagnosis of sarcopenia in COPD relies on a combination of metrics, such as muscle mass and strength; however, significant heterogeneity exists in the diagnostic thresholds and methodologies across studies. Future efforts are needed to standardize the diagnostic techniques. Future studies should aim to elucidate the pathophysiological mechanisms linking muscle degradation to COPD progression, identify biomarkers for early detection, and evaluate the efficacy of novel therapeutic modalities targeting muscle preservation and regeneration. Additionally, integrating sarcopenia management into existing COPD care frameworks will require interdisciplinary collaboration, education, and resource allocation to translate research findings into routine clinical practice effectively.

In summary, the recognition of sarcopenia as a critical factor in COPD pathophysiology and patient outcomes has transformed the landscape of disease assessment and management. By advancing standardized measurement techniques and fostering individualized therapeutic approaches, the medical community can improve the quality of life and survival of COPD patients. Continued innovation and implementation of sarcopenia-focused strategies hold promise for addressing the unmet needs in COPD care and reducing the global burden of this debilitating disease.
